# Surgical Procedures Used in the Treatment of Postoperative Acute Pancreatitis Grade C After Pancreatoduodenectomy—A Narrative Review

**DOI:** 10.3390/medicina62071337

**Published:** 2026-07-11

**Authors:** Ewa Grudzińska, Magdalena Gajda, Marek Zielonka, Wojciech Dubaj, Sławomir Mrowiec

**Affiliations:** Department of Gastrointestinal Surgery, Medical University of Silesia, 40-752 Katowice, Poland; ewa.grudzinska@sum.edu.pl (E.G.); marek.zielonka11@gmail.com (M.Z.); w.dubaj98@gmail.com (W.D.); smrowiec@sum.edu.pl (S.M.)

**Keywords:** acute pancreatitis, postoperative acute pancreatitis, pancreatic surgery, open abdomen, debridement, completion pancreatectomy

## Abstract

Postpancreatectomy acute pancreatitis grade C (PPAP-C) is the most severe grade of this complication, associated with the highest mortality rate after pancreatoduodenectomy (PD). Most PPAP-C cases require surgical intervention. However, no treatment guidelines for PPAP-C have been established. Literature reports are largely based on the coexistence of postoperative pancreatic fistula grade C (POPF-C) and PPAP-C, with similar surgical treatment methods. However, in PPAP-C, the evolution of inflammation can vary in course and severity: from necrosis limited to the anastomotic line to extensive necrosis involving the entire pancreatic parenchyma. In this narrative review, we summarize the surgical techniques proposed for PPAP-C treatment and attempt to create a decision-making algorithm for optimizing the choice of surgical treatment for PPAP-C depending on the extent of pancreatic necrosis and the patient’s general condition after PD. Our retrospective review is largely based on retrospective evidence and contains an inevitable selection bias. According to the cited literature, in cases of limited necrosis, parenchyma-sparing methods show an advantage over completion pancreatectomy. However, the complete pancreatic removal is usually performed in the initially more advanced cases or as a second choice when the pancreas-preserving methods fail. Therefore, the superiority of any approach cannot be clearly determined. More studies and uniform guidelines for optimal PPAP-C treatment are needed.

## 1. Introduction

Despite a reduction in mortality from 25% to 1.5–3% in high-volume centers [1], pancreatoduodenectomy (PD) is still associated with an increased risk of complications such as postoperative pancreatic fistula (POPF), delayed gastric emptying (DGE), hemorrhage (PPH), chyle leaks, and postpancreatectomy acute pancreatitis (PPAP) [1,2]. PPAP is defined as an acute inflammation of the pancreatic remnant, occurring after partial pancreatic resection and initiated in the early perioperative period, within the first three postoperative days [3]. The incidence of this complication ranges from 1 to 67% [4,5,6], depending on the type of surgery [6,7,8,9,10] and the adopted definition [3,11,12].

Currently, the most commonly used definition of PPAP is the International Study Group of Pancreatic Surgery (ISGPS) definition, which distinguishes three different PPAP grades defined by their clinical impact: grade A, postoperative hyperamylasemia, biochemical changes only; grade B, mild or moderate complications; and grade C, severe life-threatening complications leading to permanent organ failure lasting at least 48 h, reoperation, or death [3]. PPAP-C therefore includes the most severe and sometimes fatal cases of extensive pancreatic necrosis, which can lead to dramatic clinical scenarios, including complete destruction and disruption of the pancreatic anastomosis, which can cause sepsis, hemorrhage, and organ damage [3,8,11].

PPAP-C also increases the risk of pancreas-specific complications, especially POPF. According to the ISGPS, PPAP is considered one of the strongest independent predictors of POPF [3]. POPF grading is analogous to the PPAP grading, and POPF-C remains the most severe PD complication [13]. However, the role of PPAP in the development of POPF remains unresolved. The conditions most often, but not always, coexist, and it is unclear whether PPAP is a factor triggering POPF after PD [8] or an independent condition complicating the anastomotic leak [3,9,10].

Currently, there are no strict guidelines regarding the treatment of PPAP. Performing a surgery automatically indicates that the PPAP grade is C; however, there are no recommendations addressing the type and extent of these surgical procedures [3]. Our study aims to review the surgical methods available for the PPAP-C treatment and to create an algorithm to facilitate the decision-making regarding the type and extent of surgery. We have divided the possible surgical techniques into two groups: Pancreatic Parenchyma Preserving Methods (PPPM) and Method of Eliminating Pancreatic Parenchyma (MEPP). The close association of PPAP-C with POPF means that many techniques described below are proposed for the surgical treatment of both conditions.

This is a narrative review; therefore, the PRISMA methodology was not applied, which may have resulted in selection bias. However, we have made every effort to conduct a comprehensive literature search using PubMed and Google Scholar (using keywords such as “postoperative acute pancreatitis grade C”, “treatment in postoperative acute pancreatitis”, “postoperative pancreatic fistula grade C”, “treatment in pancreatic fistula grade C”, etc.) and included the most relevant publications (meta-analyses, retrospective cohort studies, and case reports) regarding human research published in English over 25 years (2000–2025). The analysis excluded publications on acute pancreatitis not related to pancreaticoduodenectomy, studies based on criteria for PPAP-C and POPF-C other than ISGPS, and works describing only non-surgical treatment. Publications that did not provide data on the surgical procedures used and their outcomes were also removed.

## 2. Pancreatic Parenchyma Preserving Methods (PPPM)

These methods are generally suitable for patients with a well-preserved distal pancreatic stump, with necrosis limited to the pancreaticojejunal anastomosis region.

### 2.1. Simple Drainage of the Peripancreatic Region (SD)

**Figure 1:**
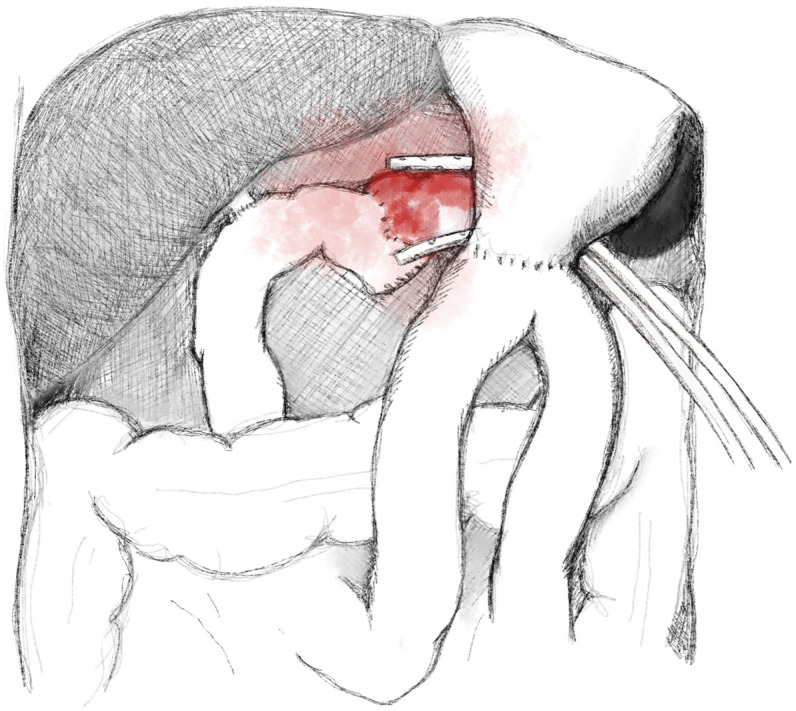
Simple drainage: two drains placed in the proximity of the pancreatojejunal anastomosis. (Inflammation marked in red in the proximity of pancreatojejunal anastomosis).

Simple drainage of the pancreatic anastomosis (SD) is the oldest and most common pancreas-sparing technique [14]. The surgery involves flushing and evacuating collections, abscesses, and pancreatic juice from the peritoneal cavity, followed by placement of drains in the proximity of the pancreatic anastomosis and/or in the area where large fluid collections were evacuated, which reduces peritoneal contamination (Figure 1). After the procedure, the abdominal cavity is either closed or the open-abdomen technique is chosen, with planned revisions of the surgical field. Drainage allows observation of the amount and type of fluid, ensuring constant monitoring and facilitating therapeutic decision-making. Furthermore, drainage can also be used for lavage of the abdominal cavity.

Mortality with the SD technique ranges from 11.1% to 67% [15,16,17,18]. The study by Zaruba [19] emphasized the safety of this strategy, resulting from its minimal invasiveness. However, this method is associated with a high risk of subsequent interventions, as the risk of further PPAP progression remains, with leakage of pancreatic juice and contamination of the peritoneal cavity, which may lead to persistent sepsis and further complications. Wroński et al. [14] emphasized that SD was associated with a higher rate of further relaparotomies (56.3%) in comparison with completion pancreatectomy (CP) (23.5%, *p* = 0.055) and external wirsungostomy (EW) (0%, *p* = 0.003). The authors emphasized that SD should be avoided in the treatment of PD complications.

The use of SD in practice is limited to a few cases of PPAP-C without obvious necrosis and without visible anastomotic damage. However, SD is most often used as one component of other PPAP-C treatment methods mentioned below, rather than as a single method, which is insufficient in cases of anastomotic necrosis. Currently, when drainage is considered a potentially sufficient intervention for pancreatitis and/or postpancreatic complications, the preferred method is percutaneous or endoscopic instead of surgical [19].

### 2.2. Pancreatojejunostomy (PJ) Separation with Closure of the Main Pancreatic Duct (MPD), Without Anastomosis Reconstruction

The goal of this method is to disconnect the jejunal loop from the pancreatic stump, ensuring separation of biliary and intestinal contents from the pancreatic juice. This, in turn, allows for the production of “pure” pancreatic juice without the activation of pancreatic enzymes, which consequently decreases pancreatic inflammation and helps treat sepsis [20].

**Figure 2:**
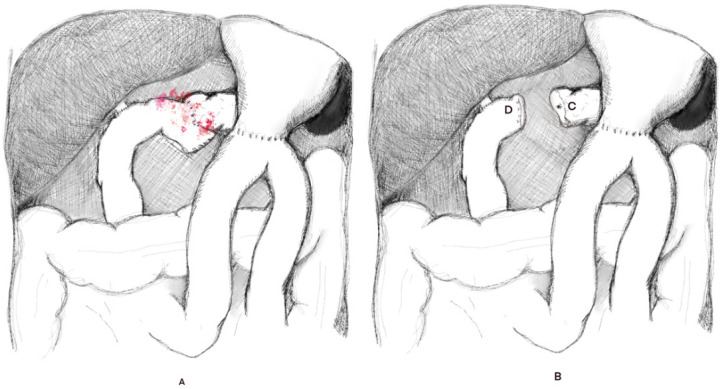
(**A**)—Inflammation marked in red in the proximity of pancreatojejunal anastomosis. (**B**)—Divided pancreatojejunal anastomosis. C—pancreatic remnant with closed MPD; D—closed jejunal loop with preserved choledochojejunostomy (CJ).

During this procedure, after separation of the pancreatojejunal anastomosis and refreshing of the end part of the intestine, the jejunum is closed proximal to the performed choledochojejunostomy (CJ). The pancreatic stump edge is also slightly shortened to obtain viable and well-perfused parenchyma. Then, the MPD is selectively closed by simple suturing—usually two layers of single monofilament non-absorbable sutures 3/0 or 4/0—fiber diameter is chosen based on the texture of the pancreatic tissues [15] or closed with cyanoacrylate or fibrin glue [17,21] (Figure 2).

Unfortunately, complications associated with this procedure include not only severe bleeding and further escalation of PPAP due to the MPD closure but also subsequent exocrine and endocrine failure due to progressive fibrosis and pancreatic atrophy [17,22,23]. In the study by Wiltberger et al., this technique led to further laparotomies in 23.1% of patients, and the mortality rate was 15.3% [20]. According to Balzano, the MPD closure technique yields similar results to SD [15]. This technique is proposed for selected patients with identifiable MPD; however, its potential benefits remain questionable compared to other MPPP methods [17,22,23].

### 2.3. Sealing the PJ—Pancreatojejunostomy Repair (PJR)

**Figure 3:**
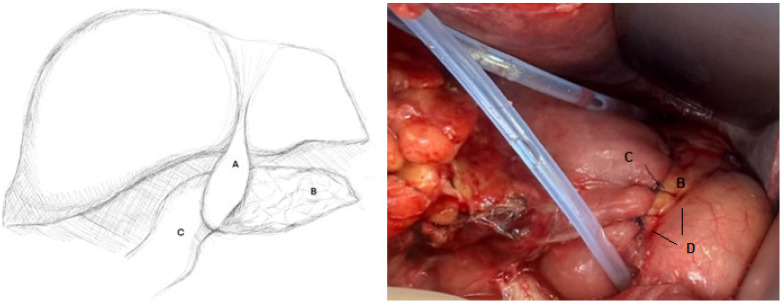
Methods of PJR. A—round ligament of the liver sealing the PJ; B—pancreatic remnant; C—jejunal loop (reconstructive); D—additional sutures sealing the PJ. Picture on the **right**—intraoperative image with new sutures sealing PJ.

PJR involves the placement of additional sutures to seal the PJ in cases of PPAP-C with minor anastomosis leakage. New sutures are placed without disconnecting the original anastomosis (Figure 3). These are typically single, monofilament, absorbable 3/0 or 4/0 sutures; the choice of suture size is based on the texture of the pancreatic tissue, with a thicker suture preferred for harder parenchyma textures. PJR has limited efficacy, and it is not routinely used in PPAP-C [14,15,23].

It is worth noting that there are other methods of sealing the anastomosis. In a case report, Egeli et al. [24] described a PJR using a round ligament of the liver, which was placed over the dehiscence site, securing both the pancreatic stump and the jejunal loop (Figure 3). During the follow-up after surgery, no postoperative complications or signs of pancreatic leakage or fistula were observed. The use of the round ligament of the liver has been previously described to protect the gastroduodenal artery (GDA) stump during PD [25], but its widespread use in PPAP-C is not described in the literature.

### 2.4. Re-Pancreatojejunostomy (Re-PJ)

Re-pancreatojejunostomy (re-PJ) in PPAP-C with anastomotic dehiscence is possible but rarely performed. During this procedure, the pancreatojejunal anastomosis is separated. The jejunal loop (reconstructive) is shortened and closed proximally to the CJ. Approximately 3 cm of the pancreatic stump must be mobilized and slightly shortened (usually by resection of ca. 1–2 cm of parenchyma) so that its edges are fresh and well-perfused. Next, a new PJ is constructed, most often as a two-layer end-to-side anastomosis with single monofilament absorbable 3/0 or 4/0 sutures. Additionally, two drains are placed in the anastomosis area.

This technique can theoretically be used in a relatively good general condition of the patient, hemodynamically stable, and able to undergo a surgery longer than, e.g., simple drainage. Sometimes, the reanastomosis is performed in a deferred manner, after the use of other methods [21,26], or as a part of other treatment methods (see Section 4).

### 2.5. Drainage of the MPD

If pancreatojejunal anastomosis leakage occurs in the course of PPAP-C, causing POPF, the pancreatic juice can damage the surrounding tissues and organs, leading to progression of pancreatitis with other possible complications (hemorrhage, perforation, sepsis, diffuse peritonitis, multi-organ failure, and death). Therefore, effective drainage of pancreatic juice into the gastrointestinal tract (internal drainage) or outside the peritoneal cavity (external drainage) may lead to better control of PPAP-C. MPD drainage methods are also appropriate for hemodynamically unstable patients who cannot undergo lengthy surgical interventions [22]. To perform these procedures, it is essential to be able to intraoperatively identify and cannulate the MPD.

#### 2.5.1. External Drainage of the MPD—External Wirsungostomy (EW)

**Figure 4:**
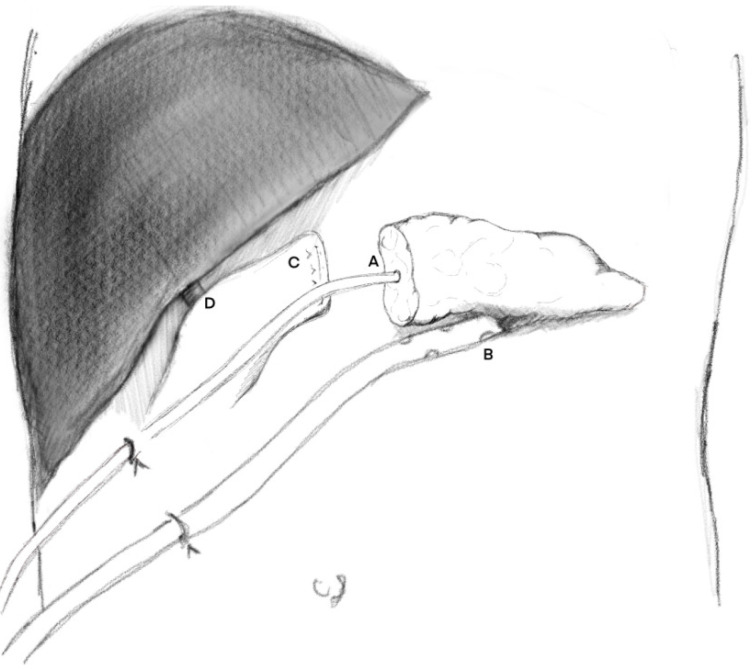
External Wirsungostomy (EW). A—proximal end of the drain placed in the MPD in the pancreatic stump; the distal end is led through the abdominal wall and secured to the skin. B—abdominal drain in the area of the pancreatic stump. C—closed jejunal loop. D—choledochojejunostomy.

During this procedure, the pancreatojejunal anastomosis is divided. The jejunal loop is shortened and closed proximally to the performed CJ. If bleeding from the pancreatic stump is detected, precise hemostasis is necessary. The next step is to insert a soft silicone drain with multiple perforations, with a diameter matching the diameter of the MPD (most often 6 Fr or 8 Fr). The drain is placed with the perforated end in the pancreatic stump (up to 2 cm deep) and secured with several single sutures. Attention must be paid to avoid occlusion of the MPD lumen. The distal end of the drain is led through the abdominal wall and secured to the skin (Figure 4). Pancreatic juice is thus drained externally and does not accumulate in the peritoneal cavity, and its quantity and appearance are monitored. This technique replaces the intraperitoneal pancreatic fistula with an extra-abdominal one, which increases control over PPAP-C and may lead to its suppression. Additionally, it is recommended to leave additional peritoneal drains (usually two) in the area of the pancreatic stump to help remove the residual pancreatic juice and any exudate from the peritoneal cavity.

It should be noted, however, that EW may be only the first stage of treatment (combined methods—see Section 4). According to some authors, the next step, after stabilizing the patient’s condition, may be re-PJ [21,26,27] or complete pancreatectomy [26]. In some patients, however, the secretion stops spontaneously, without signs of stasis and with normal endocrine function, and a simple removal of the drain is possible [21,22].

EW is a commonly used method, especially in the case of coexisting PPAP-C and POPF-C. Many studies have emphasized the undoubted advantages of this method, such as its simplicity, safety, and low mortality. In the study by Paye et al. [26], it was 17%, in the study by Horvath [21], 15%, and in the study by Ribero [27], 0%. In many patients, pancreatic endocrine function is also preserved, although this is not always the case.

However, with this method, the need for subsequent reoperations [21,22,26] should be considered, with a frequency of up to 31% [21]. After this type of intervention, patients often require prolonged hospitalization—in the study by Denost et al. [22], the median hospital stay was 42 days, in the study by Horvath [21], 58 days, and in the study by Paye et al. [26], 62 days.

#### 2.5.2. Internal Drainage of the MPD—Bridge Anastomosis

This technique also involves drainage of the MPD, but into the lumen of the gastrointestinal tract. The procedure is similar to the external drainage, but the distal end of the drain is placed inside the lumen of the first reconstructive jejunal loop and secured with sutures. This method converts an intraperitoneal pancreatic fistula to a pancreatointestinal anastomosis bridged with a drain to prevent leakage.

However, this technique is more technically challenging than EW and carries a higher risk of failure. The ongoing inflammation and gastrointestinal peristalsis can impede healing and lead to leakage and further development of PPAP and POPF. External drainage can be more closely monitored, with the type and quantity of contents monitored, enabling faster identification of a sudden decrease in pancreatic juice outflow, indicating the drain dislocation. The internal drainage dislocation is not easily spotted.

Bridge anastomosis is not a widely used technique, and literature reports are limited to small patient groups. In their study, Kent et al. [28] used this approach in five patients. All patients survived and were discharged from the hospital without complications. In the study by Ma et al. [29], the bridge technique was used as the next step in treatment after external drainage of the MPD. Xu J et al. [30] emphasized the importance of this technique in cases of massive hemorrhage—seven patients who underwent this surgery survived without exocrine or endocrine insufficiency.

Based on the available literature, it can be said that bridge anastomosis is a rarely used technique. Examples of its application are limited and require further research.

### 2.6. Conversion of the Pancreatojejunal Anastomosis to the Pancreatogastric Anastomosis (PJ to PG Conversion)

During the PD procedure, the pancreatogastric anastomosis (PG) is an alternative reconstructive method [31], although currently less frequently used.

**Figure 5:**
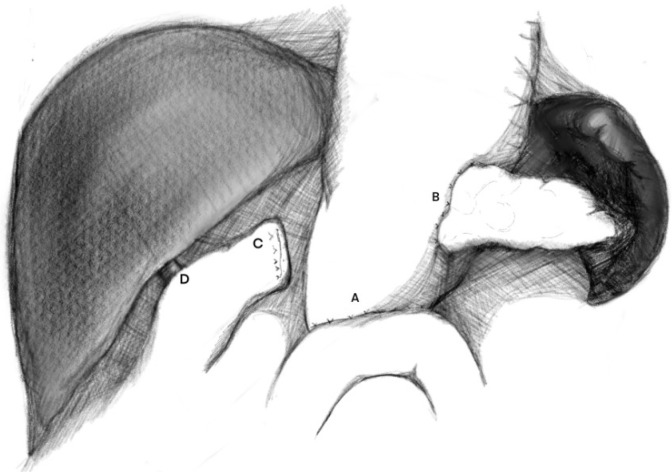
Pancreatogastrostomy. A—gastrojejunal anastomosis, B—pancreatogastrostomy, C—closed jejunal loop, D—choledochojejunostomy.

In the case of PPAP-C with anastomotic disruption, replacing the PJ with a PG is one of the methods that allows for the preservation of the pancreatic parenchyma. During this procedure, the PJ is divided. The reconstructive jejunal loop is shortened and closed proximally to the CJ. The pancreatic stump must be mobilized approximately 3 cm long and the end slightly refreshed (usually by resection of 1–2 cm of tissue). Adhesiolysis is performed to the necessary extent, and the posterior gastric wall is mobilized. These steps can be technically challenging, given that the surgical field is significantly altered by ongoing peritonitis. Next, the posterior gastric wall is incised—careful hemostasis is essential due to the abundant blood supply of the gastric mucosa. The size of the gastrostomy opening should be approximately 2–3 mm in the case of planning a duct-to-mucosa PG or larger, in the case of implantation of the entire pancreas cross-section into the gastric wall. The anastomosis is most often performed with two layers of 3/0 absorbable sutures, end-to-side (Figure 5).

Sometimes, to facilitate PG, the pancreas-to-posterior gastric wall anastomosis is performed with an approach from the gastric cavity, through an anterior gastrotomy, which is closed after the procedure is completed [32].

Postoperative gastric decompression via a nasogastric tube is recommended, facilitating efficient drainage of gastric and pancreatic juice [31].

This method can be used in the following cases:

- The patient is in relatively good general condition, allowing for the longer surgical time required for PG anastomosis with mobilization of the gastric wall, compared to drainage methods and PJ reanastomosis
- The absence of massive postoperative adhesions—anatomical conditions should allow for safe mobilization of the posterior gastric wall
- The absence of significant damage to the gastric wall caused by the ongoing inflammatory process
- The absence of significant gastric mucosal disease
- For the duct-to-mucosa technique, MPD must be identifiable intraoperatively.

The good blood supply of the gastric wall increases the chances of proper anastomotic healing, and its acidic environment prevents the activation of pancreatic enzymes, thus reducing the risk of proteolysis within the anastomosis. Good endoscopic access to the gastric cavity provides easy postoperative monitoring of the anastomosis [33]. However, at the same time, the anastomosis can result in a deficiency of pancreatic enzyme activity, leading to digestive disorders and malabsorption. A rich blood supply to the gastric wall increases the risk of postoperative bleeding [34].

Lee SJ et al. [35] reported on 181 patients who underwent PD with PJ. Six patients developed a clinically significant pancreatic fistula– two patients underwent completion pancreatectomy, and four underwent anastomosis conversion from PJ to PG. The mortality rate among patients after CP was 100%, and patients who converted from PJ to PG did not develop any significant long-term complications. Bachellier P et al. [36] described 403 patients after PD—85 underwent PJ and 318 PG. POPF-C occurred in 12 patients—all after PD with PJ. All patients required reoperation—eight patients underwent CP, and four underwent PG. Mortality in the group of patients after CP was 50%. In the group of patients who underwent rescue PG, mortality was 0%. Similar excellent survival after PG was reported by Govil et al. [18]—none of the patients who underwent rescue PG died or required additional interventions before hospital discharge.

Given the high survival rate, PG should be considered for PPAP-C, especially in hemodynamically stable patients with limited pancreatic necrosis. It must be noted, however, that in the cited studies, CP may have been chosen for the patients who were initially in worse condition, with more advanced pancreatic necrosis, causing selection bias.

## 3. Methods of Eliminating the Pancreatic Parenchyma (MEPP)

### 3.1. Debridement of the Peripancreatic Region (Damage Control)

PPAP-C usually requires debridement, which involves repeated and thorough rinsing of the surgical field with evacuation of the infected pancreatic necrosis, hemostasis, removal of fluid collections and abscesses, and placement of drains [15]. Due to the need for repeated revisions, the abdominal cavity is often not definitively closed; instead, the “open abdomen” technique is used, with systematic revisions of the pancreatic area and regular removal of necrotic tissue resulting from ongoing PPAP-C [14,21,23].

Indications for debridement include:

- Hemodynamically unstable patients, in septic shock, with metabolic acidosis, coagulopathy, poor prognosis, who would not survive more complex interventions due to their serious general condition, as a damage control technique;
- Advanced, extensive, and continuously progressing necrosis with the formation of collections and abscesses;
- Significantly altered intra-abdominal conditions with anatomical planes disrupted by the inflammation, when other surgical techniques are impossible.

The debridement of the necrotic tissues, i.e., their mechanical removal, in the case of PPAP-C, is most often associated with a laparotomy, which allows for direct control of the surgical field while ensuring the best visualization (without the step-up approach recommended in other non-postoperative types of acute pancreatitis).

The advantage of this method is its simplicity, while its disadvantage is the need for subsequent interventions (multiple irrigation, suction, drainage), which prolongs treatment time, increases costs, and increases the risk of further complications [14,21,23]. The effectiveness of this method depends on anatomical accessibility and the degree of demarcation of the necrotic lesions—difficult localization may limit complete removal of the necrosis and necessitate more invasive surgical procedures. When necrosis is not clearly separated from living tissues, the procedure can come with more complications and higher mortality.

### 3.2. Completion Pancreatectomy (CP)

Completion pancreatectomy (CP) is a procedure involving the complete removal of the pancreatic remnant with the removal of the PJ. The jejunal loop disconnected from the pancreas is shortened and closed proximally to the CJ, and, most often, simultaneous splenectomy is performed. This procedure eliminates the source of inflammation and infection, i.e., the pancreatic tissue, but it also causes inevitable exocrine and endocrine insufficiency and may lead to metabolic deterioration. Available publications suggest that relatively few patients after PD require CP (0.74–9%) and that these procedures are associated with a high mortality risk (Table 1).

Indications for CP include:

- Necrosis involving all/most of the pancreatic tissue—advanced parenchymal destruction does not allow for sparing the gland.
- Progressive necrosis spreading beyond the pancreas, into the surrounding tissues/organs, with damage and/or perforation of surrounding organs.
- Pancreatic apoplexy [42]: fulminant necrotizing pancreatitis developing after PD and leading to CP within 3 days: this phenomenon is associated with extremely high mortality (75%), and in these cases, CP often becomes the rescue treatment of choice. Pancreatic apoplexy is histologically associated with higher rates of pancreatic necrosis (p = 0.044) and hemorrhage (p = 0.001) and is accompanied by significantly higher levels of lactate dehydrogenase, C-reactive protein, serum amylase, serum lipase, drain amylase, and drain lipase compared with patients with CP after the third day [42].
- Significant disruption of the PJ continuity, preventing its safe reconstruction (suturing or reanastomosis).
- Inability to find the MPD, which is associated with the inability to effectively drain the MPD.
- Diffuse peritonitis with developing sepsis.
- Active bleeding/hemorrhage or features of previous bleeding (hematomas, anemia, sentinel bleeding).
- Lack of improvement in the patient’s general condition after the PPPMs are performed (combined methods—see Section 4).

Therefore, CP may be required in extremely severe cases of PPAP-C. In the studies listed in Table 1, the most common indications for CP were POPF -C and PPAP-C (necrotic acute pancreatitis or pancreatic ischemia).

Patients after CP procedures have varying mortality rates, ranging from 14.3% to 75%. This could be due to the very different overall condition of patients after CP. It is worth noting that the higher mortality after CP procedures may therefore result more from patient selection itself (life-threatening complications: septic shock, hemorrhage) than from the weakness of the method itself.

Destruction caused by inflammation and necrosis significantly worsens intraoperative conditions, altering the anatomy of the pancreatoduodenal region, which prolongs the procedure and increases intraoperative blood loss. Resection of the remaining pancreas might be extremely challenging in the inflamed and fragile surgical field. Numerous reports indicate that, given the destruction caused by necrosis, CP is technically very difficult and carries an increased risk of complications and mortality. Loos et al. [37] studied 3953 patients after PD procedures, of whom 120 (3%) required CP, most often due to necrotic PPAP (*n* = 47, 39%). The median CP time in this study was 165 min (IQR, 130–235 min), and the median blood loss during CP was 1450 mL (IQR, 500–3000 mL). In the study by Garnier J et al. [44], the median blood loss was 600 mL, and the median operative time was 240 min. These data underscore the difficulty and complexity of this procedure. A meta-analysis by Groen et al. [41], which analyzed 33 studies involving 745 patients, confirmed the association between CP and mortality (OR = 1.99; 95% CI = 1.03–3.84). In their analysis, the authors compared PPPM with CP and concluded by emphasizing the advantages of pancreas-sparing procedures.

Although CP is an aggressive procedure, potentially preventing the need for further surgeries, the need for a subsequent laparotomy cannot be ruled out. In Bressan et al.’s [45] analysis of 11 studies, re-laparotomy following CP was required in 35% of patients. However, it should be noted that Balzano et al. [15] emphasized that patients undergoing CP required a repeat laparotomy less frequently than patients with preserved pancreas (7% vs. 59%, *p* < 0.01). Similar conclusions were reached by Wroński et al. [14]– in their study, simple drainage was associated with a higher rate of further relaparotomies (56.3%) compared with completed pancreatectomy (23.5%, *p* = 0.055) and external wirsungostomy (0%, *p* = 0.003).

A natural consequence of CP is the development of postoperative exocrine and endocrine insufficiency. The absolute insulin deficiency caused by CP leads to the development of brittle, hard-to-manage, insulin-dependent diabetes (type 3c), where blood sugar levels can be more unstable, hypoglycemia can be more severe and harder to recognize, and treatment requires especially careful monitoring [15]. For type 3c diabetes, insulin therapy with regular blood sugar monitoring is essential, along with diet control (regular meals, adjusting insulin doses to the amount of carbohydrates eaten, and being careful with exercise due to the risk of hypoglycemia) and simultaneous treatment of exocrine pancreatic insufficiency (taking pancreatic enzymes with meals, monitoring nutritional status, and checking levels of fat-soluble vitamins).

However, when comparing type 3c diabetes with type 1 diabetes, many authors point out the lack of significant differences. Roberts KJ et al. [48] noted that while CP was associated with significant mortality risk and a reduced overall quality of life compared to a matched control group of type 1 diabetes patients who had not had surgery, the diabetic condition that occurred after CP did not seem to differ significantly from the condition in the control group of diabetic patients when assessed according to diabetes-specific outcomes. Similar results were reported by Jethwa et al. [49]—diabetes after CP does not necessarily mean bad blood sugar control and, in most cases, leads to comparable biochemical control compared to the normal population of people with type 1 diabetes.

Some authors point to the possibility of autologous pancreatic islet transplantation (AIT) for better glycemic control [15,50,51], but only after the patients’ overall condition stabilizes. In work by Balzano et al. [15], AIT was associated with CP in seven patients, of whom one died post-operatively. Long-term graft function was maintained in four out of six surviving patients, with one insulin-independent patient at 36 months after transplantation. Authors emphasized that AIT can reduce the metabolic impact of the CP.

It should be remembered that during reoperations after PD, the integrity of the remaining anastomoses should be checked: the choledochojejunostomy and the gastrojejunostomy. Unfortunately, in cases of severe necrotizing pancreatitis, the remaining anastomoses are often damaged, and the procedure should then be extended to include additional surgical procedures (including drainage or reconstruction of the remaining anastomoses).

According to the available data and based on our experience, we believe that the CP procedure should be a last resort (salvage method) when conservative procedures are not possible [37,39,41,43], due to the high postoperative mortality rates, which can be up to 75% (Table 1). Furthermore, considering the risks and technical challenges, this procedure should be performed by an experienced surgical team, and performing it as an emergency procedure outside the day’s working hours should be avoided [45].

### 3.3. Near-Completion Pancreatectomy (NCP)

Near-complete pancreatectomy (NCP) is a technique most commonly used in the treatment of severe, chronic pancreatitis in the end stage when other treatment methods have failed, often to alleviate chronic pain [19]. In the case of PPAP—C, NCP is performed as a substitute for CP when, due to difficult postoperative conditions caused by progressive necrosis, inflammation, and postoperative adhesions, access to the peripheral portion of the pancreas may be very difficult, if not impossible. Splenectomy in conditions of disrupted anatomy and the inability to clearly identify the surrounding vessels can pose an extremely high risk of potentially fatal intraoperative hemorrhage. In such circumstances, preservation of the residual pancreatic tissue (a portion of the pancreatic tail) is considered acceptable. Furthermore, sparing even a small fragment of the pancreas may potentially allow for at least partial preservation of exocrine and endocrine function. During NCP, the PJ and most of the pancreatic parenchyma (approximately 90–95%) are removed, leaving a small part of the pancreatic tail near the splenic hilum [52].

However, it should be remembered that leaving residual parenchyma may lead to further progression of PPAP and possible complications (including hemorrhage, perforation, and postoperative splenic damage). Vigilant observation of the patient is necessary, and in the event of further complications, reoperation and splenectomy with removal of the remaining pancreatic tissue (e.g., via an incision in the left subcostal region) are necessary.

A summary of the works cited in Chapters 2 and 3, along with the most important conclusions, can be found in Table S1 in the Supplement.

## 4. Combined Methods

It is not always possible to limit the intervention to just one treatment method for PPAP-C. The complexity and severity of this complication often require the use of multiple methods simultaneously and/or radically change the initial treatment strategy. Sometimes, treatment begins with PPPM, but due to a lack of response, MEPP is ultimately used [26].

Possible sequences of treatment methods for PPAP—C, according to the literature:

- EW → bridge anastomosis [29];
- EW → re-PJ [21,26,27];
- EW → CP [26];
- SD → CP (rescue CP after failed SD was associated with extremely high mortality rates) [14].

## 5. Minimally Invasive Methods for Treating PPAP-C

Our narrative review mainly focuses on surgical methods involving classic laparotomy. A patient’s severe general condition and the need for immediate treatment rarely allow for a step-up approach, which is common in classic acute pancreatitis. Minimally invasive methods most often combine debridement with simple drainage—they do not allow for more complex procedures like PJR, re-PJ, drainage of the MPD, PJ to PG conversion, CP, and NCP. Patients in poor overall condition, with extensive necrosis, sepsis, diffuse peritonitis, and active bleeding, require complex interventions through classic laparotomy.

Minimally invasive methods can, however, be used in cases of well-defined pancreatic necrosis (walled-off necrosis—WON) and pseudocysts in patients who are generally stable. They are easy to repeat and usually form part of multi-step treatment—a single procedure is often not enough and needs to be repeated. They can also serve as the first step before more invasive procedures. A summary of the types of minimally invasive treatments can be found in Table 2.

## 6. PPPM vs. MEPP—What to Choose?

Many authors emphasize that PPPM is more beneficial for patients than MEPP [37,39,41,43]. Preserving even a small part of the pancreas can prevent or alleviate endocrine and exocrine insufficiency. Diabetes, developing after CP, is particularly burdensome for the patient due to its unstable course and difficult-to-control glycemia, with a high tendency for sudden glycemic fluctuations and high insulin requirements [59]. In the Balzano et al. study, diabetes was significantly more common in patients after CP than in those after PPPMs (91% vs. 42%, *p* = 0.017) [15].

Furthermore, the PPPM reoperations are less invasive for the patients compared to MEPPs. Fewer technical difficulties and shorter procedure time translate into lower postoperative mortality, as reported by many authors [37,39,41,43]. However, it should not be forgotten that pancreas-sparing methods are characterized by a higher tendency for further surgical interventions. In the study by Balzano et al. [15], pancreas-sparing treatment was associated with a significantly higher rate of relaparotomy compared to CP (59% vs. 7%, *p* = 0.003).

In their study, Zhou et al. [60] reported that the rates of repeat relaparotomy, mortality, and endocrine failure were 31.8%, 42%, and 100% for CP, 25%, 21.3%, and 17.8% for PJ dissection with preservation of the pancreatic stump, 10.4%, 14.9%, and 12.3% for internal or external wirsungostomy, 12.5%, 0%, and 25% for rescue PG, and 30%, 47.9%, and 12.5% for SD. In their conclusions, the authors emphasize that PPPMs should be the preferred treatment option.

It should be clearly emphasized, however, that MEPP methods are usually used in the most difficult clinical situations, in patients in extremely severe general condition, in cases of extensive necrosis, sepsis, bleeding, or after unsuccessful previous interventions. The higher mortality associated with MEPP methods, especially with CP, may therefore reflect patient selection and disease severity, rather than the weakness of the procedure itself.

A summary of the advantages and disadvantages of each treatment method is presented in Table 3.

**Figure 6:**
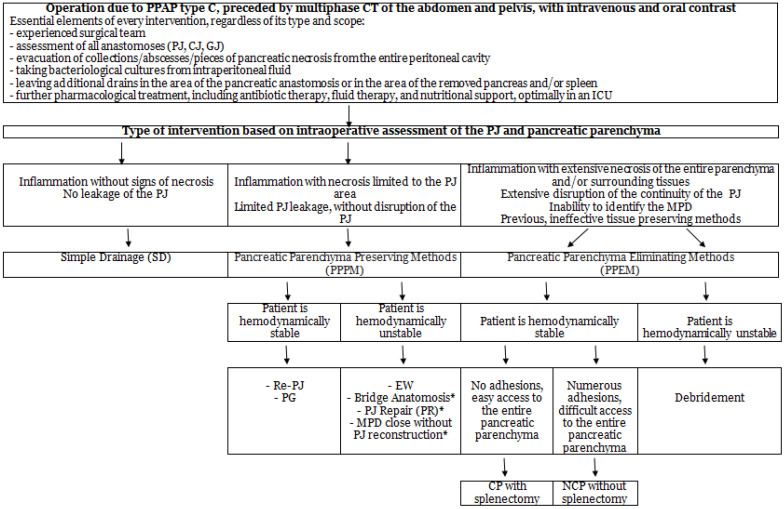
Proposed treatment algorithm for PPAP—C depending on the patient’s general condition and the extent of necrosis. The algorithm is hypothesis-generating and requires external verification because of unavoidable selection bias. * methods rarely used, the literature reports are based on small groups of patients.

Based on our narrative review, the superiority of any approach (PPPM or MEPP) cannot be clearly determined. In our decision-making algorithm (Figure 6), we propose selecting a treatment method based on an assessment of the patient’s general condition and the extent of pancreatic necrosis. While the assessment of the patient’s general condition is based on commonly available scales, such as Acute Physiology and Chronic Health Evaluation II or The Sequential Organ Failure Assessment, assessing the extent of necrosis during relaparotomy can be a significant challenge for the surgeon, prone to the risk of error. In their study, Wroński et al. [14] emphasized that enzymatic necrosis within the pancreatic parenchyma was found in all CP specimens, although in half of the cases the gland was considered viable intraoperatively. Preoperative imaging diagnostics has a crucial role– multiphase computed tomography (CT) of the abdomen and pelvis with intravenous and oral contrast. In our previous studies [61,62], diffuse or localized inflammatory enlargement of the pancreatic stump, inflammatory changes in the peripancreatic fat tissue, peripancreatic fluid collections, necrosis of the pancreatic parenchyma, peripancreatic necrosis, leakage of pancreatic anastomosis, peritonitis, and bleeding were significantly associated with the development of PPAP (*p* < 0.001). Additionally, intraoperative assessment of the surgical field using indocyanine green may help in an objective assessment of the vascularization of the pancreatic stump, thus facilitating the decision regarding the extent of the procedure [63].

We also recommend that reoperation due to PPAP-C should be performed, whenever possible, by an experienced surgical team specializing in pancreatic surgery, in order to optimally select the type of treatment and avoid technical errors [45]. Furthermore, early reoperation is associated with reduced Intensive Care Unit (ICU) stay [62]. In the Wiltberger study [20], the median postoperative day of reoperation was 11 days after the first surgery (2–24), but the patients who underwent reoperation within 10 days after surgery had a significantly shorter operation and recovery time in the ICU.

Regardless of the treatment method used, during reoperation, the following rules should be observed:

- Evacuation of all collections, abscesses, and necrotic masses by repeated irrigation of the surgical site
- Thorough hemostasis, with particular attention to the pancreatic tissue
- Inspection of all anastomoses (PJ, CJ, GJ)
- Placement of abdominal drains—at least two, one proximal and one distal to the PJ; additional drains may be necessary in areas following evacuation of large collections, removal of the spleen, etc.
- Culture collection from the peritoneal fluid for bacteriological testing to enable targeted antibiotic therapy.

## 7. Study Limitations

A few limitations of this narrative review need to be considered, as they may affect the scope and interpretation of the conclusions presented. PPAP-C is a relatively newly defined phenomenon, and the methods to treat it are not clearly established. In our work, we tried to organize the available surgical methods, creating an algorithm that is hypothesis-generating and requires external validation in further studies as well as development within a formal consensus. The algorithm we propose is therefore only conceptual—it is not a verified decision-making tool, just a suggestion. In our algorithm, we did not include minimally invasive methods, whose role in treating PPAP-C is limited.

The available evidence is mostly based on retrospective series, small cohorts, and case reports. Due to the narrative nature of the review, no formal assessment of the methodological quality of all included studies was conducted, which may increase the risk of subjectivity in interpreting the results. In addition, in our analysis, we excluded studies based on definitions of POPF and PPAP other than those from ISGPS, which may have led to the omission of some important research. Additionally, most of the studies we cited focused mainly on treating POPF-C, rather than directly on PPAP-C, which is a disrupting factor.

Because of these limitations, the results should be interpreted with caution, and confirming them requires further research, especially high-quality, multi-center prospective studies.

## 8. Conclusions

PPAP-C is a severe complication following PD, often coexisting with pancreatojejunal anastomosis leakage. The causes and treatment methods for this complication are not fully determined. The literature on PPAP-C management is based on small groups and retrospective studies, and currently, there is no clear consensus. In our opinion, based on the current literature, if surgery is needed, the selection of the appropriate treatment method should depend on the severity of pancreatic necrosis, the patient’s general condition, and the surgeon’s experience. In most publications, the use of pancreas-sparing methods is recommended due to less metabolic disruption and lower mortality. Elimination of the pancreatic tissue is usually chosen as the last resort when pancreas-preserving methods fail or when the necrosis is extensive. They are associated with high postoperative mortality; however, being usually performed for more advanced cases, or as a second choice in previously reoperated patients, it can not be determined that they are less effective in general. Since most conclusions in our work are based on retrospective studies and our review is subject to an inevitable selection bias, further high-quality, multi-center prospective studies and international guidelines for the management of PPAP-C are necessary to enable optimal treatment.

## Figures and Tables

**Table 1:** Incidence and mortality in completion pancreatectomy after PD.

Author	Year	CP After PD (%)	Mortality (%)
Wroński M et al. [14]	2019	2.7	47.1
Balzano et al. [15]	2014	2	21.4
Ribero D et al. [27]	2013	6.2	43.5
Loos M et al. [37]	2023	3	37
Nentwich MF et al. [38]	2015	3.8	55
Gueroult S et al. [39]	2004	2.8	38
Tamijmarane A et al. [40]	2006	3.7	52
Groen JV et al. [41]	2021	0.74	56
Mintziras I et al. [42]	2022	5.4	75 * or 36 ** (with * or without ** pancreatic apoplexy)
Bramis K et al. [43]	2023	-	40
Garnier J et al. [44]	2021	4.6	23.8
Bressan AK et al. [45]	2018	3	42
Almond et al. [46]	2015	3	52.6
Smits FJ et al. [47]	2017	9	54

**Table 2:** Minimally invasive methods proposed for treating PPAP-C, according to the step-up approach.

Type of Method	Method Description
1. Percutaneous drainage under imaging guidance (USG/CT) [53,54]	It involves inserting a catheter into a fluid collection or necrosis to eliminate infected pancreatic necrosis and remove fluid collections and abscesses. It requires precise planning of the access path based on a CT/USG scan, assessing the relationship to blood vessels and abdominal organs, in order to avoid damaging internal organs and anatomical structures As far as technical possibilities allow, passing through the peritoneal cavity is avoided, preferring an extraperitoneal approach It most often serves as the first stage of treatment before further procedures.
2. Endoscopic Drainage [55,56]	Performed during an endoscopy, most often under the guidance of endoscopic ultrasound (EUS). It involves creating a connection between the stomach or duodenum and a fluid reservoir. Under EUS guidance, the spot with the shortest distance to the reservoir is chosen, avoiding large blood vessels. A puncture and widening of the access channel are performed, then a draining prosthesis is placed.
3. ndoscopic Debridement [55,56]	It is usually performed after prior drainage and creating an access channel to the necrosis. It is typically implemented when endoscopic drainage alone does not provide sufficient clinical improvement, or when the collection is dominated by a solid component. It involves introducing a gastroscope through an inserted stent or a mature fistula into the cavity, followed by mechanically removing necrotic tissue under visual control using endoscopic tools. Most often, the procedure needs to be repeated several times later to fully eliminate the necrotic tissue.
4. Video-Assisted Retroperitoneal Debridement (VARD) [57,58]	A hybrid minimally invasive technique that combines a small surgical access point with the use of laparoscopic tools to directly visualize and remove necrotic tissue using the retroperitoneal space. The procedure starts by gaining access through a small, lateral incision along the lumbar line, through which laparoscopic instruments and optics are introduced, and then the necrotic tissue is removed, usually without the need for a laparotomy. Allows direct control of the surgical field and mechanical removal of necrosis while limiting soft tissue trauma, reducing the risk of abdominal contamination and systemic complications compared to a classic laparotomy.

**Table 3:** Summary of advantages and disadvantages of PPAP-C treatment methods. * Selection bias may also play a significant role in the results: PPPM assignment is a non-random assignment, potentially favoring healthier patients for PPPM.

Type of Intervention	Advantages	Disadvantages
Pancreatic parenchyma preserving methods (PPPM)	Main advantage: possible preservation of some of the endocrine and exocrine functions by leaving part of the gland Shorter operation time [14] Less invasive procedure [19,21] Lower mortality rate *—yes [18,21,27,35,36]; no [14] Technically easier [21] Less frequent multiorgan failure [14]	Main disadvantage: lack of definitive elimination of the PPAP-C source, i.e., the inflamed pancreatic tissue Frequent need for repeated interventions, both planned (anastomotic reconstruction in the case of external drainage [14,27]) or unplanned (deterioration of the general and local condition caused by further evolution of PPAP [20])
Methods eliminating the pancreatic parenchyma (MEPP)	Main advantage: definitive elimination of the PPAP-C cause Less frequent reoperations—yes [15]; no [60]	Main disadvantage: imminent exocrine and endocrine failure (brittle diabetes) Longer operation time [14] More demanding technically [14] More invasive Higher mortality rate *—yes [21]; no [14] More frequent blood transfusions [14]

## Data Availability

No new data were created or analyzed in this study.

## Supplementary Materials

The following supporting information can be downloaded at: https://www.mdpi.com/article/10.3390/medicina62071337/s1. Table S1: Overview of the studies cited in the article.

## Abbreviations

The following abbreviations are used in this manuscript:

AIT	Autologous Islet Transplantation
CJ	Choledochojejunostomy
CP	Completion Pancreatectomy
CT	Computed Tomography
DGE	Delayed Gastric Emptying
EUS	Endoscopic Ultrasonography
GDA	Gastroduodenal Artery
EW	External Wirsungostomy
GJ	Gastrojejunostomy
ICU	Intensive Care Unit
ISGPS	International Study Group of pancreatic Surgery
MEPP	Method of Eliminating Pancreatic Parenchyma
MPD	Main Pancreatic Duct
PPPM	Pancreatic Parenchyma Preserving Method
NCP	Near Completion Pancreatectomy
PD	Pancreatoduodenectomy
PG	Pancreatogastrostomy
PJ	Pancreatojejunostomy
PJR	Pancreatojejunostomy Repair
POPF	Postoperative Fistula
PPAP	Postpancreatectomy Acute Pancreatitis
PPH	Postpancreatectomy Hemorrhage
re-PJ	Re-pancreatojejunostomy
SD	Simple Drainage
USG	Ultrasonography
WON	Walled-Off Necrosis
VARD	Video-Assisted Retroperitoneal Debridement
