# Supplementary material for: Surgical Procedures Used in the Treatment of Postoperative Acute Pancreatitis Grade C After Pancreatoduodenectomy—A Narrative Review
Source: Medicina (Kaunas). 2026 Jul 11;62(7):1337. doi: 10.3390/medicina62071337 (PMC13413669; doi:10.3390/medicina62071337)
Supplement: Supplementary file 1 [file medicina-62-01337-s001.zip › medicina-4351475-supplementary.pdf]

Author	Year	Type of article	Aim of the study	Reoperation due to POPF-C	Occurrence of PPAP-C features in the operation field*	Number of patients require surgical treatment after PD	Surgical technique	Mortality [%]	Need for another reintervention	Endocrine/exocrine insufficiency
Wroński M et al. [14]	2019	Retrospective cohort	Compare the outcome of CP, EW, and SD in the treatment of grade C postoperative pancreatic fistula	Yes	Yes	43	CP (n=17) vs. EW (n=10) vs. SD (n=16)	CP: 47.1 vs. EW: 50 (P = 0.883) vs. SD: 56.3 (P = 0.598)	SD was associated with a higher rate of further relaparotomies (56.3%) in comparison with CP (23.5%, P= 0.055) and EW (0%, P = 0.003)	NI
Balzano et al. [15]	2014	Retrospective cohort	Compare the outcome of PPPM with CP in the treatment of postoperative pancreatic fistula	Yes	No	31	CP (n=14) vs. PPPM (n=17)	CP: 21.4 vs. PPPM: 29.4 (*P*=0.610)	Patients undergoing a CP required a further relaparotomy less frequently than patients with PPPM (7% versus 59%, *P* < 0.01)	Insulin-dependent diabetes was more frequent in patients treated with CP (10/11, 91% vs. 5/12, 42%, *P* = 0.017). Seven patients (78%) successfully received AIT
de Castro SMM et. al [16]	2005	Retrospective cohort	Management of pancreatic fistula after PD	Yes	Yes	27	SD (n=8) PPPM other than SD (n=10) CP (n=9)	SD: 25 (n=2) PPPM other than SD: 30 (n=3) CP: 0 (n=0)	SD (n=2) PPPM other than SD (n=3) CP (n=2)	All patients who underwent CP developed pancreatic endocrine insufficiency, compared with three of seven patients after PPPM other than SD
Govil S et al. [18]	2012	Retrospective cohort	Salvage PG after PD	Yes	NI	12	PG (n=4) CP(n=2) SD (n=6)	PG: 0 (n=0) CP: 50 (n=1) SD: 66 (n=4)	PG (n=4) CP(n=2) SD (n=6)	Diabetes: PG (n=1) CP(n=1) SD (n=1)
Haddad LBP et al. [17]	2009	Retrospective cohort	Management of pancreatic fistula after PD	Yes	Yes	14	CP (n=5); Debridement and drainage (n=8); other (n=1)	CP: 40 (n=2); Debridement and drainage: 12.5 (n=1)	NI	NI
Záruba P et al. [19]	2022	Retrospective cohort	A comparison of surgical approaches in the treatment of grade C postoperative pancreatic fistula	Yes	NI	54	SD (n=18); disconnections of the PJ (n=28); CP (n=8)	The 90-day mortality was 46% vs. 6% vs. 38% in the PJ disconnection group, the SD	SD (n=2.9) disconnections of the PJ (n=6.7) CP (n=5.6)	NI

								and the CP group, respectively ($p = 0.013$)		
Horvath P et al. [21]	2016	Retrospective cohort	Pancreas-preserving surgical management of grade-C pancreatic fistulas after pancreatic head resection by EW	Yes	Yes	13	EW (n=12) SD (n=1)	17 (n=2)	31% (n=4)	NI
Denost Q et al. [22]	2012	Retrospective cohort	Wirsungostomy as a salvage procedure after PD	Yes	Yes	21	EW (n=21) (also CP: n=2, but these patients were not included for analysis)	28.5 (n=6)	4.7% (n=1)	Diabetes: n=3 Exocrine insufficiency: n=15
Egeli T et al. [24]	2016	Case Report	Round ligament of the liver as a patch for reparation of leakage of the PJ	Yes	Yes	1	Methods of PJR: round ligament of the liver sealing the PJ	No	No	NI
Paye F et al. [26]	2013	Retrospective cohort	Evaluation of the effects of treating POPF using EW followed by PJ reconstruction	Yes	Yes	21	EW (n=12) CP (n=4)	EW:17	Yes[1] – planned, repeat PJ in 10 patients	EW – n=4, but long-term endocrine function was unaltered in 66% of patients who benefited from EW
Ribero D et al. [27]	2013	Retrospective cohort	Compare the outcome of PPPM with CP in the treatment of postoperative pancreatic fistula	Yes	NI	42	PPPM(n=19) [EW (n=9) + other various procedures (n=10)] vs. CP (n=23)	CP: 43.5 vs. PPPM: 0	CP: 39.1 vs. PPPM: 11.1	None of the PPPM patients developed diabetes
Ma T et al. [29]	2018	Retrospective cohort	Evaluation of the effects of treating POPF using EW followed by PJ reconstruction in the treatment of postoperative pancreatic fistula	Yes	NI	11	EW (n=10)	40	Yes[1] – planned, repeat PJ in 3 patients	None of the patients with EW and repeat PJ developed endocrine and exocrine deficiency
Königsrainer I et al. [32]	2010	Retrospective cohort	Evaluation of the effects of treating POPF using EW followed by PJ	Yes	NI	4	EW	25	Yes[1] – planned, repeat PJ in 3 patients	NI

			reconstruction in the treatment of postoperative pancreatic fistula							
Lee SJ et al [35]	2022	Retrospective cohort	Analysis of the results after PJ to PG conversion in the treatment of postoperative pancreatic fistula	Yes	NI	6	PJ to PG conversion (n=4) CP (n=2)	PJ to PG conversion: 0 CP:100	No	None of the patients with PJ to PG conversion developed new endocrine deficiency
Loos M et al. [37]	2023	Retrospective cohort	Identification the indications for and report the outcomes of CP	Yes	Yes	120	CP	37	NI	NI
Nentwich MF et al. [38]	2015	Retrospective cohort	Analysis of the results after CP in the treatment of for post-pancreatic surgery complications	Yes	Yes	20	CP	55	Yes, CP (n=5)	NI
Gueroult S et al. [39]	2004	Retrospective cohort	Analysis of the outcomes after CP for postoperative peritonitis	Yes	Yes	8	CP	38	Yes: CP (n=3)	Yes: problems with brittle diabetes (n=4) and (n=1) lack of pancreatic enzymes tolerance.
Tamijmarane A et al. [40]	2006	Retrospective cohort +	Analysis of results after CP for post-pancreatic surgical complications	Yes	Yes	25	CP	52	Yes: CP (n=3)	Yes: all survived patients (n=12) develop diabetes
Groen JV et al. [41]	2021	Multicentre cohort study and meta-analysis	Comparison of CP with a PPPM in the treatment of postoperative pancreatic fistula	Yes	NI	162	CP (n=36) PPPM (n=126)	CP: 56 PPPM: 31.7	Yes : CP (n=6), PPPM (n=21)	Yes: CP – NI; PPPM - endocrine insufficiency (n=19), and exocrine insufficiency (n=32)
Mintziras I et al. [42]	2022	Retrospective cohort	Comparison of CP procedures performed within 3 days after PD (n=8) to procedures done after 3 days (n=25) after PD	Yes	Yes	33	CP	75 (with pancreatic apoplexy) or 36 (without pancreatic apoplexy)		Yes
Bramis K et al. [43]	2023	Retrospective cohort	Analysis of results after CP for complications following PD	Yes	NI	10	CP	40	NI	NI

Garnier J et al. [44]	2021	Retrospective cohort	Four-step standardized technique used in CP for grade C postoperative pancreatic fistula after PD	Yes	NI	30	CP (n=21)<br>Others (n=9)	CP: 23.8	CP: 23.8% (n=5)	NI
Bressan AK et al. [45]	2018	Systematic Review	Analysis of results after CP in the treatment of postoperative pancreatic fistula	Yes	NI	151	CP	42	CP: 35%	NI
Smits FJ et al. [47]	2017	Retrospective cohort	Management of severe pancreatic fistula after PD	Yes	Yes	64	CP: 17<br>Others: 47	35.9	NI	Endocrine insufficiency (n=22), and exocrine insufficiency (n=22)
Zhou Y-M et al [60]	2018	Systematic Review	Comparison of surgical procedures performed due to pancreatic fistula after PD	Yes	Yes	370	SD (n=48)<br>EW/IW (n=55)<br>CP (n=200)<br>PJ to PG conversion (n=8)<br>Disconnection of PJ (n=47)	SD: 47.9<br>EW/IW: 14.9<br>CP: 42<br>PJ to PG: 0 conversion<br>disconnection of PJ: 21.3	SD: 30<br>EW/IW: 10.4%<br>CP: 31.8%<br>PJ to PG conversion: 12.5%<br>disconnection of PJ: 25%	Long-term endocrine insufficiency :<br>SD: 12.5%<br>EW/IW: 12.3%<br>CP: 100%<br>PJ to PG conversion: 25%<br>disconnection of PJ: 17.8%

Table S.1 Overview of the studies cited in the article. CP = completion pancreatectomy, EW = external wirsungostomy; NI = no information; PPPM = Pancreatic Parenchyma Preserving Method; AIT = Autologous Islet Transplantation; em-CP = emergency completion pancreatectomy; el-CP = elective completion pancreatectomy; PD = pancreatoduodenectomy ; PJ = pancreatojejunostomy; PG = pancreatogastrostomy

* - due to the lack of a clear definition of PPAP-C, many studies have described features typical for PPAP-C in the operation field, such as inflammation and necrotic tissues

+ - the work concerned, besides PD, also other pancreatic surgeries, like distal pancreatectomy

[1] – a planned follow-up surgery, which is the next step in the scheduled surgical treatment
